# A Multi-AI Agent Framework for Interactive Neurosurgical Education and Evaluation: From Vignettes to Virtual Conversations

**DOI:** 10.1227/neuprac.0000000000000217

**Published:** 2026-03-13

**Authors:** Karl L. Sangwon, Jeff Zhang, Robert Steele, Jaden Stryker, Joanne J. Choi, Jin Vivian Lee, Daniel Alexander Alber, Aly Valliani, Nivedha Kannapadi, James Ryoo, Austin Feng, Hammad A. Khan, Sean Neifert, Cordelia Orillac, Hannah K. Weiss, Nora C. Kim, David Kurland, Howard A. Riina, Douglas Kondziolka, Michal Mankowski, Eric Karl Oermann

**Affiliations:** 1 Department of Neurological Surgery, NYU Langone Health, New York, New York, USA;; 2 Department of Surgery, NYU Langone Health, New York, New York, USA;; 3 Department of Radiology, NYU Langone Health, New York, New York, USA;; 4 Center for Data Science, New York University, New York, New York, USA;; 5 Neuroscience Institute, NYU Langone Health, New York, New York, USA

**Keywords:** Large language models, Artificial intelligence, Neurosurgical education

## Abstract

**BACKGROUND AND OBJECTIVES:**

Traditional medical board examinations present clinical information in static vignettes with multiple-choices (MC), fundamentally different from how physicians gather and integrate data in practice. Recent advances in large language models (LLMs) offer promising approaches to creating more realistic clinical interactive conversations. However, these approaches are limited in neurosurgery, where patient communication capacity varies significantly and diagnosis heavily relies on objective data such as imaging and neurological examinations. We aimed to develop and evaluate a multi–artificial intelligence (AI) agent conversation framework for neurosurgical case assessment that enables realistic clinical interactions through simulated patients and structured access to objective clinical data.

**METHODS:**

We developed a framework to convert 608 Self-Assessment in Neurological Surgery first-order diagnosis questions into conversation sessions using 3 specialized AI agents: patient AI for subjective information, system AI for objective data, and clinical AI for diagnostic reasoning. We evaluated generative pretrained transformer 4o's (GPT-4o's) diagnostic accuracy across traditional vignettes, patient-only conversations, and patient + system AI interactions, with human benchmark testing from 10 neurosurgery residents.

**RESULTS:**

GPT-4o showed significant performance drops from traditional vignettes to conversational formats in both MC (89.0%-60.9%, *P* < .0001) and free-response scenarios (78.4%-30.3%, *P* < .0001). Adding access to objective data through system AI improved performance (to 67.4%, *P* = .0015; and 61.8%, *P* < .0001, respectively). Questions requiring image interpretation showed similar patterns but lower accuracy. Residents outperformed GPT-4o in free-response conversations (70.0% vs 28.3%, *P* = .0030) using fewer interactions and reported high educational value of the interactive format.

**CONCLUSION:**

This multi-AI agent framework provides both a more challenging evaluation method for LLMs and an engaging educational tool for neurosurgical training. The significant performance drops in conversational formats suggest that traditional MC testing may overestimate LLMs' clinical reasoning capabilities, while the framework's interactive nature offers promising applications for enhancing medical education.

**ABBREVIATIONS:**
AI: artificial intelligence
CNS: Congress of Neurological Surgeons
CRAFT-MD: Conversational Reasoning Assessment Framework for Testing in Medicine
FR: free-response
GPT-4o: generative pretrained transformer 4o
LLMs: large language models
MC: multiple-choice
PGY: postgraduate year
SANS: Self-Assessment in Neurological Surgery.

Clinical diagnosis in neurosurgery requires gathering and integrating diverse clinical data: patient history and examination findings, diagnostic studies, and temporal progression of symptoms.^1^ In clinical practice, physicians must gather information through focused questions and diagnostic testing—an active process that static board preparation materials have difficulty simulating.^2-4^ Although traditional vignette-style questions effectively test clinical reasoning through carefully constructed scenarios, such as the Self-Assessment in Neurological Surgery (SANS) question bank, they present all information upfront—fundamentally different from how physicians obtain and integrate clinical data in actual practice.^5^

Creating interactive educational experiences that better reflect clinical decision-making—such as oral board examinations or standardized patient encounters—demands substantial faculty resources.^6^ Recent advances in artificial intelligence (AI) have enabled new approaches to this challenge.^7-9^ Although large language models (LLMs) such as generative pretrained transformer-4 (GPT-4) have demonstrated strong performance on medical board examinations,^8-11^ achieving passing scores on neurosurgery certification tests,^12-14^ the Conversational Reasoning Assessment Framework for Testing in Medicine (CRAFT-MD)^15^ revealed potential limitations in their abilities. CRAFT-MD used LLMs to transform static vignettes into interactive conversations with a simulated patient AI. In this framework, GPT-4 performed significantly worse on MedQA–USMLE diagnostic questions compared with standard multiple-choice (MC) format—suggesting that conversational formats may better challenge and accurately evaluate diagnostic reasoning abilities.^15,16^

However, the original CRAFT-MD framework's single-agent approach has limitations in neurosurgical scenarios. Many neurosurgical patients cannot effectively communicate because of altered consciousness, and critical diagnostic information often comes from sources beyond patient history—detailed neurological examinations, multimodal imaging, laboratory studies, and neurophysiological testing.

To address these limitations, we present a multi-AI agent framework for neurosurgical case assessment (Figure 1) that enables interaction with both simulated patients and objective clinical data. Our framework introduces 3 specialized AI agents: (1) a patient AI that provides subjective information and simulates varying levels of consciousness and communication ability; (2) a system AI that manages access to objective clinical data, including imagings, laboratory results, and examination findings; and (3) a clinical AI that synthesizes information for diagnostic reasoning. This multi-AI agent approach enables the clinical user to actively gather both subjective and objective information, better reflecting actual diagnostic practice in neurosurgery.

**FIGURE 1.**
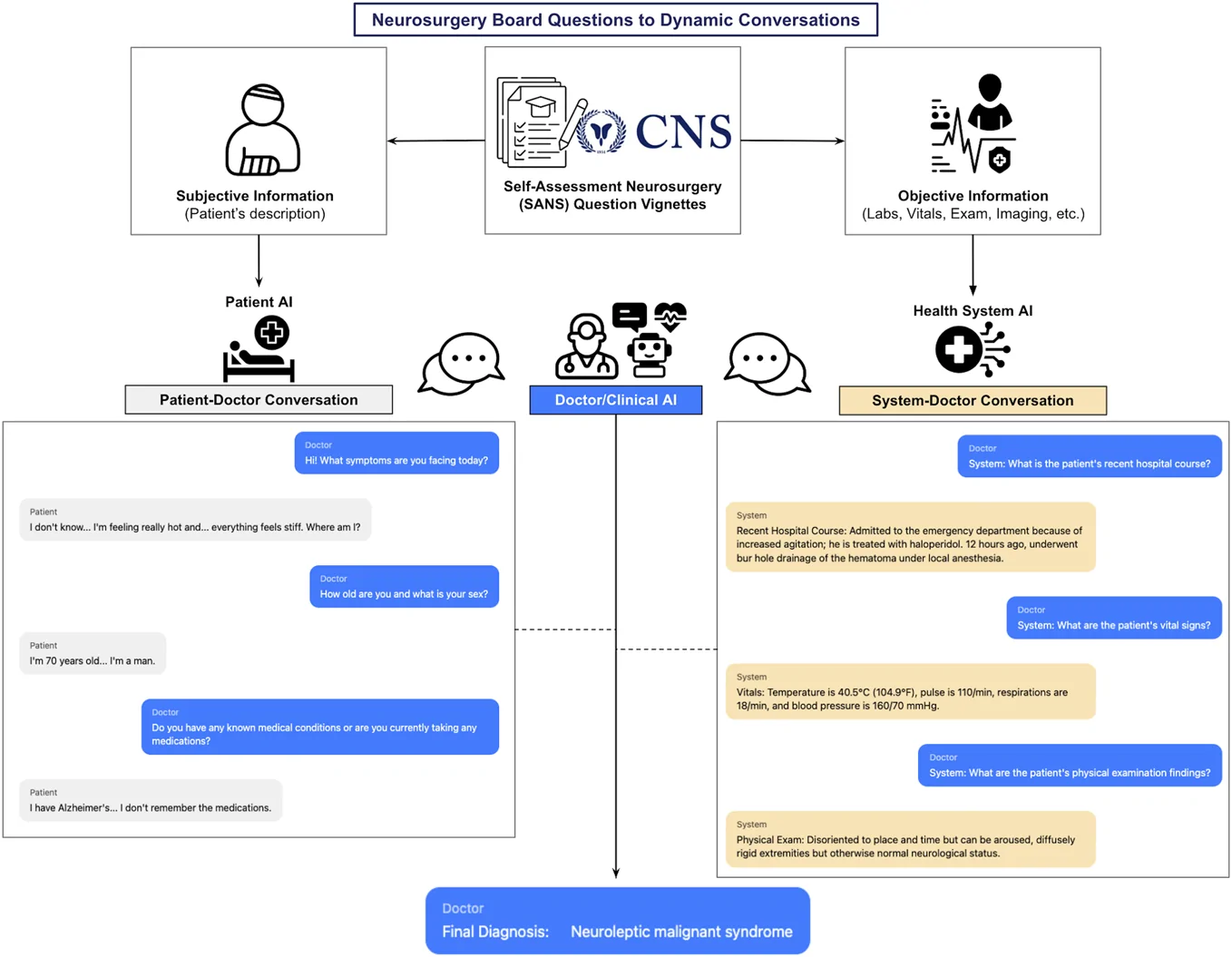
Multi-AI agent framework for converting board-style questions to interactive clinical scenarios. The framework transforms SANS board questions into dynamic conversations by separating information into subjective (patient-reported) and objective (clinical data) components. A clinical AI or doctor navigates 2 distinct conversational interactions: (1) patient-doctor conversation through a patient AI that provides subjective information based on the patient's description, and (2) system-doctor conversation through a health system AI that supplies objective clinical data (laboratory results, vital signs, examination findings, and imaging). The clinical AI or human user integrates information from both channels to formulate a final diagnosis. Example conversation snippets demonstrate the natural flow of history-taking and clinical data gathering through these parallel interfaces. AI, artificial intelligence; CNS, Congress of Neurological Surgeons. *CNS logo © 2025 Congress of Neurological Surgeons. All rights reserved*.

We evaluate this framework across multiple formats through testing with generative pretrained transformer 4o (GPT-4o) and neurosurgery residents, comparing traditional vignettes, patient AI-only conversations, and patient + system AI interactions. This work advances both LLM evaluation methods and educational tools in neurosurgery, offering insights into the effectiveness of different assessment formats and the potential of AI-assisted training approaches.

## METHODS

### Data Source and Question Selection

#### Institutional Review Board and Legal

This project was institutional review board–approved (i23-00510) and reviewed by Congress of Neurological Surgeons (CNS) leadership. Patient consent was not required as no patients were involved.

#### Question Inclusion and Exclusion Criteria

We processed 3895 SANS questions with GPT-4o to identify 608 first-order diagnosis questions as inclusion criteria. These included questions on primary diagnosis, lesion localization, or pathology, excluding those that test next steps or higher-order reasoning. Six hundred eight questions were further divided into 218 text-based diagnosis or 390 image-based diagnosis questions based on presence of image files associated with the original question vignette. Image-based diagnosis questions were separated to evaluate on the questions that relied primarily on correct interpretation of the provided images (eg, radiologic scans) (Figure 2A).

FIGURE 2.Study design and GPT-4o performance analysis. **A**, Flowchart depicting the selection and processing of SANS ABNS questions. From 3985 total questions, 608 first-order diagnosis questions were identified and categorized into text-based (n = 218) and image-based (n = 390) sets. Questions were presented in either original vignette or conversation framework format, with MC or FR options. Responses were assessed by an evaluator AI. **B**, GPT-4o diagnostic accuracy on text-based questions (n = 218, 5 trials each) across different formats. Performance significantly improved with system AI integration compared with patient AI alone (*P* < .0001 for MC, *P* = .0015 for FR). **C**, GPT-4o diagnostic accuracy on image-based questions (n = 390, 5 trials each), showing similar patterns of improvement with system AI integration (*P* = .0643 for MC, *P* < .0001 for FR). Error bars represent 95% CIs. Dotted lines represent 25% random chance baseline for MC questions. ABNS, American Board of Neurological Surgery; AI, artificial intelligence; CNS, Congress of Neurological Surgeons; FR, free-response; GPT-4o, generative pretrained transformer 4o; LLM, large language model; MC, multiple-choice. *Figure 2A, CNS logo © 2025 Congress of Neurological Surgeons. All rights reserved.*

**Figure:**
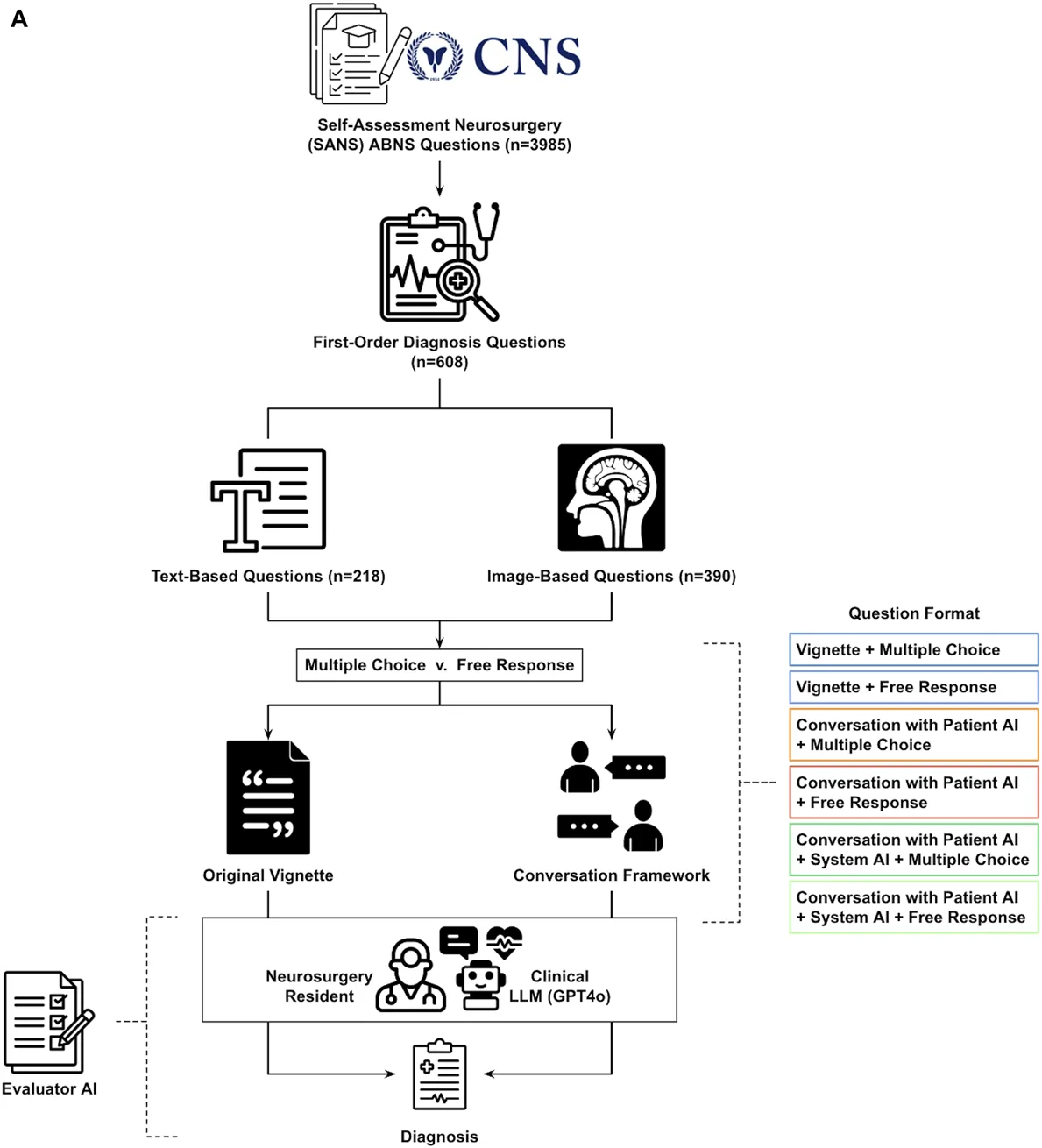

**Figure:**
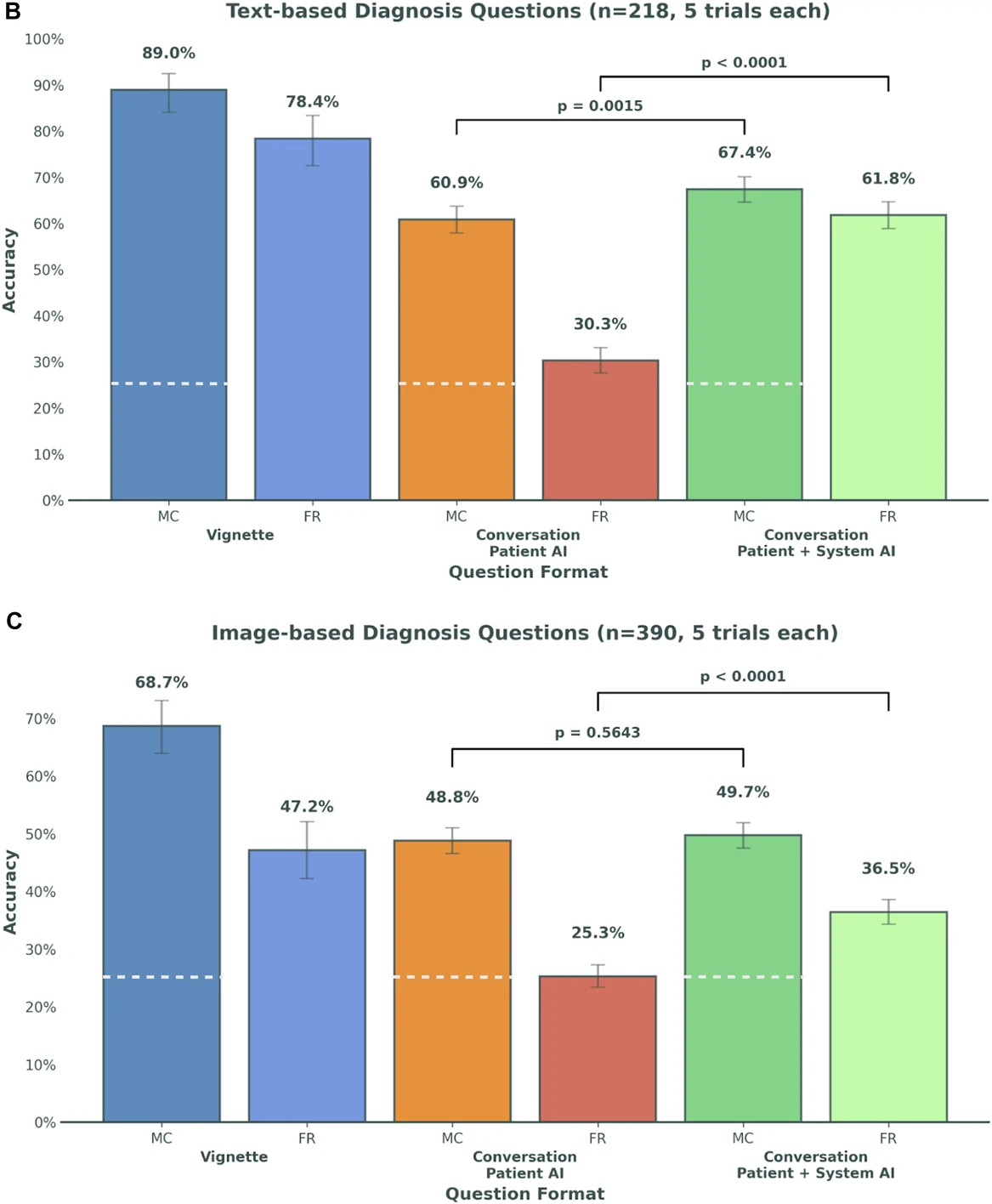

### Data Availability

SANS questions were used with permission of the CNS and will not be made publicly available as they are intellectual property of the CNS.

### Multi-AI Agent Conversation Framework Architecture

We developed a novel multi-AI agent framework for neurosurgical case assessment that was inspired by CRAFT-MD.^15^ The framework was implemented using the NYU Langone Health-specific instance of GPT-4o as the foundational LLM, deployed via a Health Insurance Portability and Accountability Act–compliant Azure environment. GPT-4o’s temperature parameter was set to 0.1 for response consistency. All model development and evaluation was performed in Python version 3.10.

Our framework comprises 3 distinct AI agents: clinical AI, patient AI, and system AI. Each agent serves a specialized function in the diagnostic process, with clinical AI handling diagnostic reasoning, patient AI providing subjective information, and system AI managing objective information. Detailed prompts for all AI agents and the evaluation system are provided in **Supplemental Digital Content 1** (**Supplemental Table**, http://links.lww.com/NS9/A90).

#### Clinical AI Agent

The clinical AI agent was implemented in 3 formats:

##### Question Format: Vignette

In this format, we maintained the traditional SANS question format, processing questions with or without MC [ie, “free-response (FR)”].

##### Question Format: Conversation With Patient AI Only

For conversation-style SANS questions based on the original CRAFT-MD framework, the clinical AI was prompted to conduct patient interviews through structured, single-line questions.

##### Question Format: Conversation With Patient + System AI

Upon upgrading our conversation framework to involve multiple agents including system AI to handle objective information from vignettes, we modified our prompt and enabled the clinical AI to alternate between patient communication and objective data acquisition through standardized “System:” queries.

#### Patient AI Agent

We implemented the patient AI in 2 formats: one following the original CRAFT-MD framework, and another with expanded response guidelines for our multi-AI agent framework.

##### Question Format: Conversation With Patient AI Only

For conversation-style SANS questions based on the original CRAFT-MD framework, the patient AI was designed to simulate patient responses using colloquial language while maintaining strict adherence to the case information.

##### Question Format: Conversation With Patient + System AI

In our framework, the patient AI agent was engineered to represent the subjective component of the clinical encounter. The response design incorporates 4 components: (1) consciousness assessment protocols for handling altered mental status scenarios; (2) age-appropriate communication guideline; (3) security protocols to maintain response integrity; and (4) standardized response parameters to ensure appropriate information disclosure. Sample patient responses under varying responsiveness are shown in **Supplemental Digital Content 2** (**Supplemental Figure 1**, http://links.lww.com/NS9/A86).

#### System AI Agent

The System AI agent was developed to function as an objective data repository, managing structured clinical information, including laboratory values, radiographic findings, physical examination data, vital signs, and hospital course documentation. This agent responds to specific queries prefixed with “System:” from the clinical AI, providing strictly objective information from the case data.

### Evaluation

We evaluated diagnostic accuracy across different question formats using both LLM and human testing. Each format was tested using the same set of SANS questions, divided between text-based and image-based diagnosis questions, with and without MC options.

#### Question Grading

For LLM, each question was processed through 5 trials to account for response variability. For MC, responses were automatically scored based on letter choice matching. For FR, responses were evaluated using a separate GPT-4o instance (evaluator AI), with established reliability^16^ for medical FR grading, to check for diagnostic equivalence. The complete evaluator AI prompt with detailed equivalence criteria and examples is provided in **Supplemental Digital Content 1** (**Supplemental Table**, http://links.lww.com/NS9/A90).

#### Human Benchmark Testing

We recruited 10 neurosurgery residents (postgraduate year [PGY]2-7) from NYU Langone Health for comparative benchmark under our new multi-AI agent conversation framework, replacing the clinical AI agent. Two each from PGY-2,3,4,5,7 were recruited. Each resident completed 8 randomly selected test-set questions (2 per format), through a standardized user interface implemented in Python using IPython widgets (Figure 2A). Each resident was provided with 30 minutes for 8 conversation sessions total. Before start, each resident was also provided a tutorial to interact with the user interface and instructed to determine the best diagnosis by interacting with both the patient AI and system AI. The interface presented cases sequentially, with format order randomized to minimize learning effects. Answer responses and complete conversation logs were collected for each case. Qualitative resident experience feedback was collected via post-test interview.

### Statistical Analysis

Diagnostic accuracy was calculated as the proportion of correct responses for each format. We computed 95% CIs using the Wilson score interval method. For comparisons between different conversation formats, we used Mann–Whitney *U* tests to assess statistical significance. Conversation length was extracted from each interaction log and reported as mean ± SD. Statistical comparisons between groups were performed using Mann–Whitney *U* tests, with effect sizes calculated using Cohen's d. For human testing, we analyzed interresident variability and format-specific performance patterns.

## RESULTS

### Patient AI-Only Conversation Framework Is Limited in Application to Neurosurgical Scenarios

Analysis of patient-only conversations showed key limitations of the CRAFT-MD framework in neurosurgical scenarios. Clinical AI frequently omitted neurological examinations, and patient AI communicated unrealistically in scenarios involving altered consciousness. In text-based questions, 73.1% contained objective findings, yet clinical AI requested them in only 32.8% and 30.3% of cases for MC and FR questions, respectively. In addition, 11.1% of cases involved inappropriate patient AI communication despite clinical contexts suggesting inability to communicate (**Supplemental Digital Content 3**, **Supplemental Figure 2**, http://links.lww.com/NS9/A87). To address these limitations, we developed and evaluated a multiagent framework incorporating access to objective clinical data via System AI.

### GPT-4o's Reduced Accuracy in Conversational Formats Improves with Objective Data Access via Multi-AI Agent Framework

In text-based SANS questions (n = 218), GPT-4o's diagnostic accuracy varied across formats. With MC, accuracy was 89.0% (95% CI: 84.1%-92.5%) for original vignettes, decreased to 60.9% (58.0%-63.8%) in patient-only conversations (*P* < .0001), and improved to 67.4% (64.6%-70.1%) with patient + system AI conversation (*P* < .0015). In FR, accuracy was 78.4% (72.5%-83.4%) for vignettes, 30.3% (27.6%-33.1%) for patient-only conversations (*P* < .0001), and increased to 61.8% (58.9%-64.7%) with patient + system AI conversation (*P* < .0001) (Figure 2B, Table 1). GPT-4o's performance in conversational MC formats remained well above the random chance baseline of 25% accuracy. FR formats have no defined numerical chance level; therefore, we treated responses from uninstructed GPT-4o as the random baseline, which by default responded without diagnostic specificity or proper clinical language format yielding 0% accuracy. Across all formats, GPT-4o surpassed this random baseline by producing consistently interpretable diagnostic statements. For image-based SANS questions (n = 390), identical patterns emerged, but all accuracies were lower than text-based questions respectively (*P* < .0001) (Figure 2C, Table 1).

**TABLE 1.** Comprehensive Performance Analysis of GPT-4o on Self-Assessment in Neurological Surgery Neurosurgery Questions

User	Clinical AI: GPT-4o											
Question type	Text-based questions (n = 218, 5 trials each)						Image-based questions (n = 390, 5 trials each)					
Question format	Vignette		Conversation with patient AI only (CRAFT-MD)		Conversation with patient + system AI		Vignette		Conversation with patient AI (CRAFT-MD)		Conversation with patient + system AI	
	MC	FR	MC	FR	MC	FR	MC	FR	MC	FR	MC	FR
Accuracy (95% CI)	89.0% (84.1%-92.5%)	78.4% (72.5%-83.4%)	60.9% (58.0%-63.8%)	30.3% (27.6%-33.1%)	67.4% (64.6%-70.1%)	61.8% (58.9%-64.7%)	68.7% (64.0%-73.1%)	47.2% (42.3%-52.1%)	48.8% (46.6%-51.0%)	25.3% (23.4%-27.3%)	49.7% (47.5%-52.0%)	36.5% (34.4%-38.6%)
Conversation length (mean ± SD)	—	—	16.73 ± 3.88	23.60 ± 5.58	18.04 ± 5.01	22.82 ± 5.85	—	—	16.19 ± 3.90	21.51 ± 4.90	17.06 ± 3.46	19.80 ± 4.54

AI, artificial intelligence; CRAFT-MD, conversational reasoning assessment framework for testing in medicine; FR, free-response; GPT-4o, generative pretrained transformer 4o; MC, multiple-choice.

Evaluation across 3 presentation formats (standard vignette, conversation with patient AI, and conversation with patient + system AI) on both text-based (n = 218) and image-based (n = 390) diagnostic questions. Each question was tested 5 times to account for response variability. Performance metrics include diagnostic accuracy with 95% Wilson score CIs and conversation length analysis (mean ± SD of message counts). MC format evaluated letter-choice accuracy, while FR format responses were assessed by an evaluator AI for diagnostic equivalence with reference answers. Conversation lengths were not applicable (—) for vignette format as these were direct question-answer pairs. All conversations were conducted through standardized protocols as detailed in Methods.

### Removing MC Options Increases Length of Conversations

**FIGURE 3.**
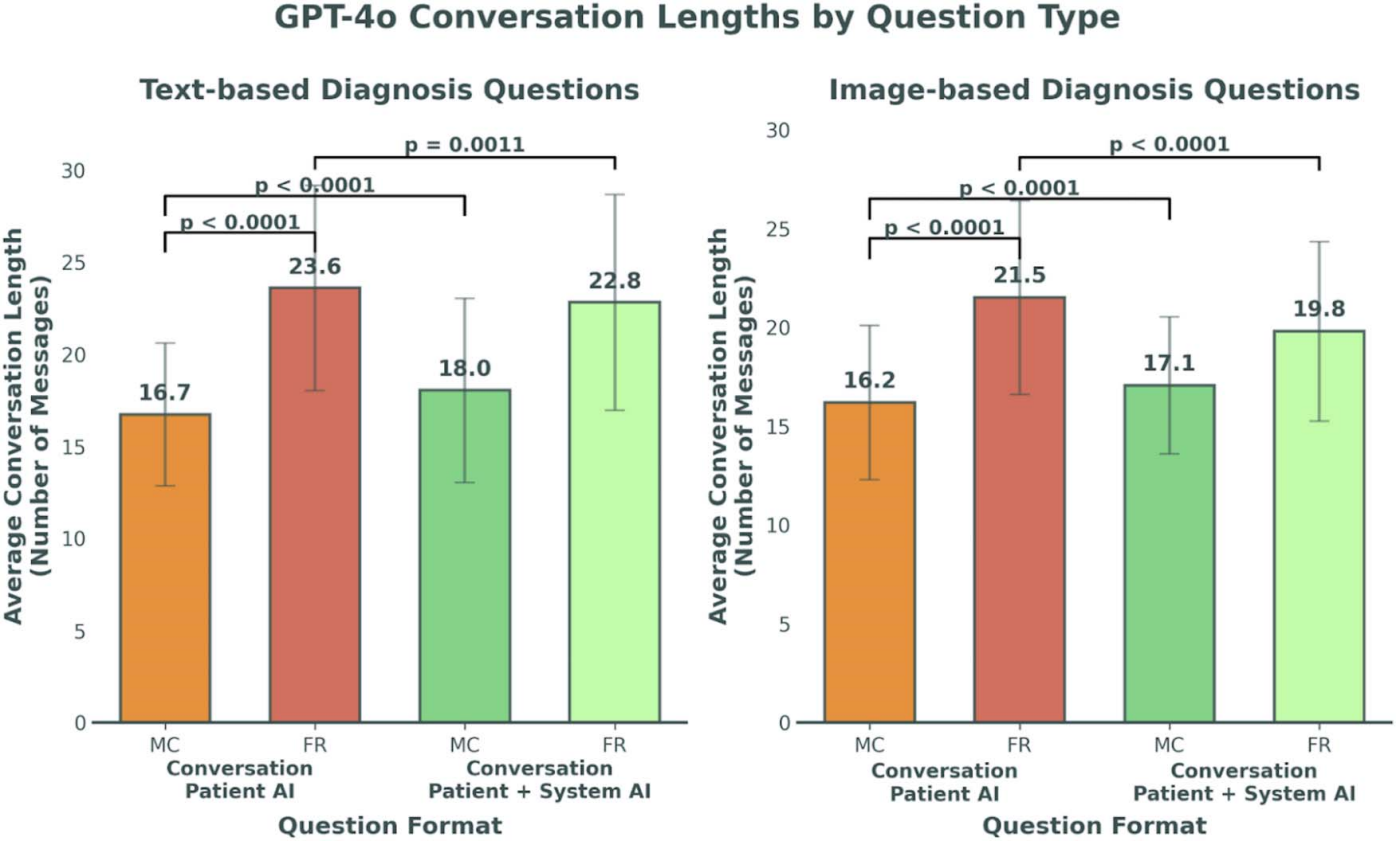
Analysis of GPT-4o conversation lengths across question formats. Average number of conversational exchanges for text-based (left, n = 218) and image-based (right, n = 390) diagnosis questions. Free-response formats consistently required more exchanges than multiple-choice formats across both patient AI-only and patient + system AI conversations (primary: 23.6 ± 5.6 vs 16.7 ± 3.9 messages, d = −1.43; image: 21.5 ± 4.9 vs 16.2 ± 3.9 messages, d = −1.20; all *P* < .0001). Integration of system AI showed small but significant effects on conversation length: slight increases in multiple-choice formats (primary: 18.0 ± 5.0 vs 16.7 ± 3.9 messages, d = −0.29; image: 17.1 ± 3.5 vs 16.2 ± 3.9 messages, d = −0.24) and decreases in free-response formats (primary: 22.8 ± 5.9 vs 23.6 ± 5.6 messages, d = 0.14; image: 19.8 ± 4.5 vs 21.5 ± 4.9 messages, d = 0.36; all *P* < .001). Error bars represent one SD. AI, artificial intelligence; GPT-4o, generative pretrained transformer 4o.

FR formats consistently required longer conversation length than MC formats across both question types (*P* < .0001). For text-based questions, patient-only conversations averaged 16.7 ± 3.9 messages (MC) and 23.6 ± 5.6 messages (FR). Patient + system AI conversations showed similar patterns: 18.0 ± 5.0 messages (MC) and 22.8 ± 5.9 messages (FR). Image-based questions followed comparable patterns (Figure 3, Table 1).

### Neurosurgery Residents Outperform LLMs and Are More Efficient at Diagnosis in FR Conversations

**FIGURE 4.**
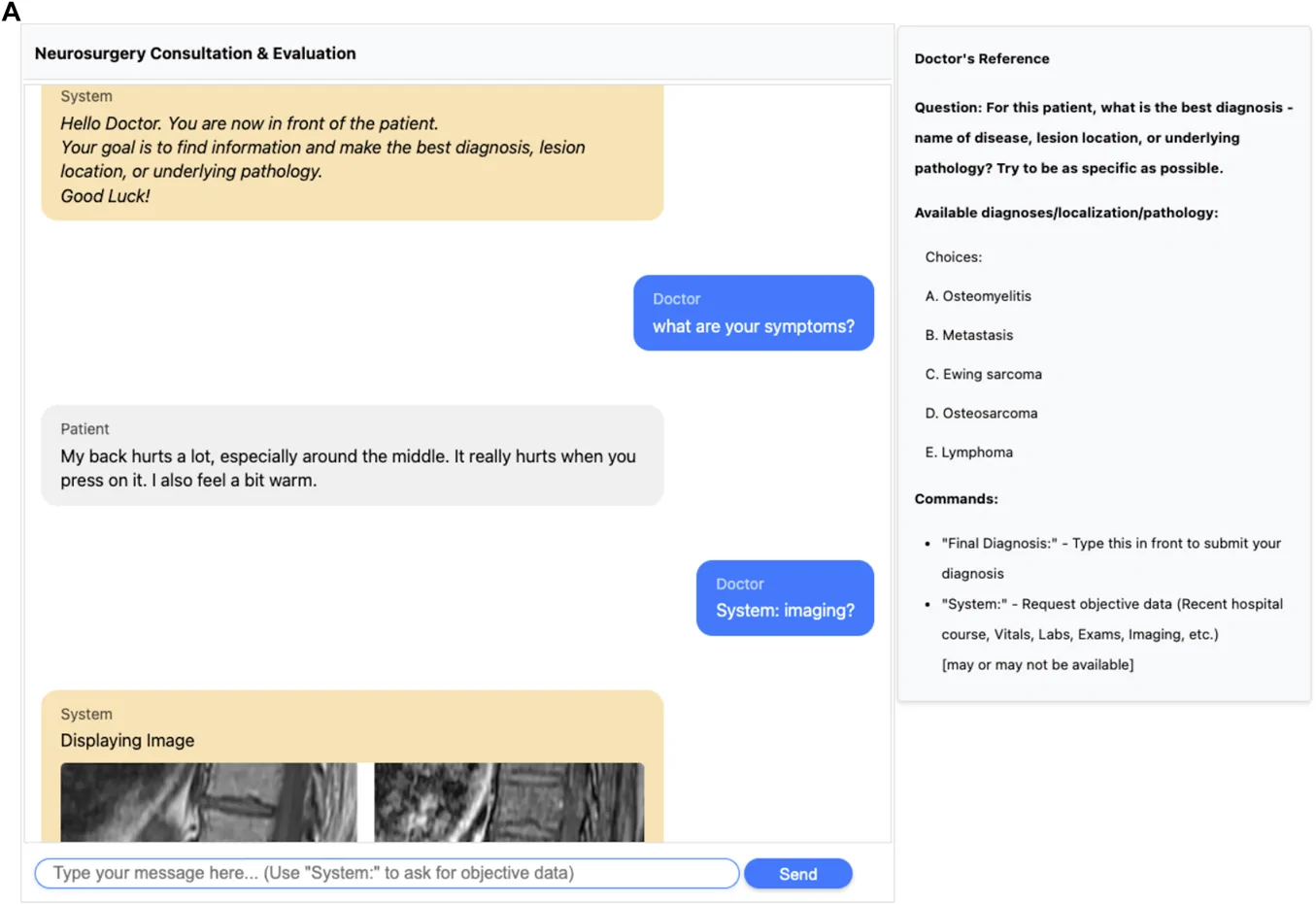

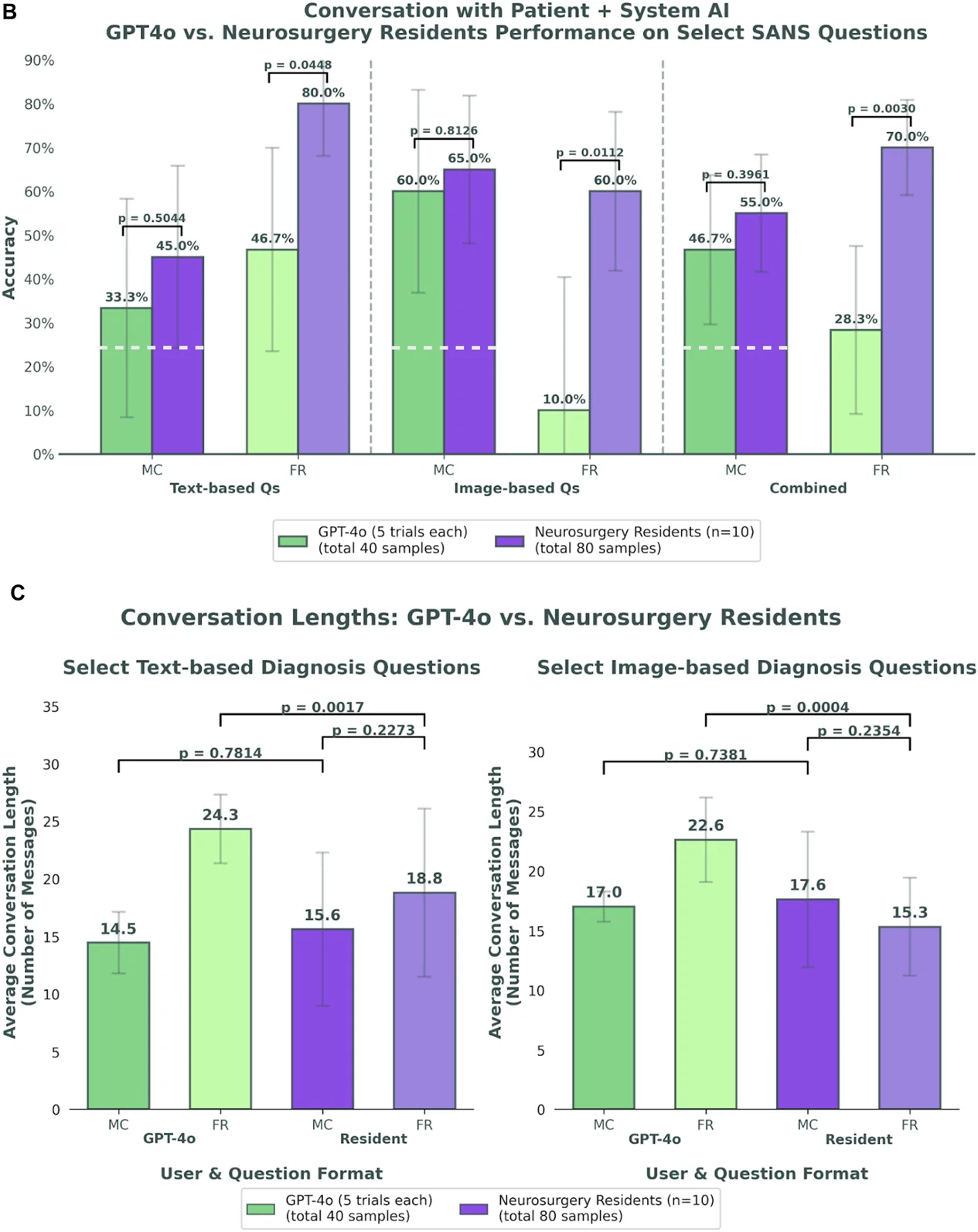
User interface and performance analysis of multi-AI agent conversation framework. **A**, The user interface demonstrates the interactive diagnostic process, where users (blue chat bubble) can engage with both patient AI (grey chat bubble) for subjective information and system AI (yellow chat bubble) for objective data (including imaging). Doctor's reference panel (right) displays the question prompt and available commands. **B**, Diagnostic accuracy comparison between GPT-4o (5 trials each, total 40 samples, green) and neurosurgery residents (n = 10, total 80 samples, purple) on select SANS questions. Results are shown separately for text-based and image-based questions, as well as combined performance, in both MC and FR formats. Error bars represent 95% CIs. **C**, Analysis of conversation lengths comparing GPT-4o and neurosurgery residents, separated by question type (text-based and image-based) and format (MC and FR). Average conversation length represents the number of messages during the diagnostic process. Statistical comparisons were performed using Mann–Whitney *U* test, with *P*-values shown for significant differences. Error bars represent one SD. Dotted lines represent 25% random chance baseline for MC questions. AI, artificial intelligence; FR, free-response; GPT-4o, generative pretrained transformer 4o; MC, multiple-choice; SANS, Self-Assessment in Neurological Surgery.

Ten neurosurgery residents (PGY2-7) collectively outperformed GPT-4o on test-set 8 SANS questions (Figure 4A), particularly in the FR format [70.0% (59.2%-80.8%) vs 28.3% (9.2%-47.5%), *P* = .0030] (Figure 4B). This difference was most pronounced in image-based questions [60.0% (41.9%-78.1%) vs 10.0% (−20.4% to 40.4%), *P* = .0112]. Residents also demonstrated greater efficiency, using 5.5 and 7.3 fewer average turns than GPT-4o for text-based and image-based FR questions, respectively (*P* = .0017, *P* = .0004). However, resident accuracy in the MC formats did not exceed GPT-4o performance. In MC questions, residents and the model performed comparably, underscoring that the constrained structure of MC items continues to advantage LLMs by reducing diagnostic uncertainty. Full comparative performance metrics are shown in Figures 4B and 4C, and Table 2.

**TABLE 2.** Comparative Analysis of GPT-4o and Neurosurgery Resident Physicians Performance on Select Self-Assessment in Neurological Surgery Neurosurgery Questions

User	Clinical AI: GPT-4o (total 40 samples)				Neurosurgery residents (n = 10; total 80 samples)			
Question type	Text-based questions (n = 4, 5 trials each)		Image-based questions (n = 4, 5 trials each)		Text-based questions (n = 4, 1 trials each)		Image-based questions (n = 4, 1 trial each)	
Question format	Conversation with patient + system AI							
	MC	FR	MC	FR	MC	FR	MC	FR
Accuracy (95% CI)	33.3% (8.4%-58.3%)	46.7% (23.4%-69.9%)	60.0% (36.8%-83.2%)	10.0% (−20.4% to 40.4%)	45.0% (24.2%-65.8%)	80.0% (68.1%-91.9%)	65.0% (48.1%-81.9%)	60.0% (41.9%-78.1%)
Conversation length (mean ± SD)	14.47 ± 2.68	24.33 ± 2.98	17.00 ± 1.26	22.60 ± 3.56	15.62 ± 6.65	18.80 ± 7.29	17.60 ± 5.70	15.30 ± 4.11

AI, artificial intelligence; FR, free-response; GPT-4o, generative pretrained transformer 4o; MC, multiple-choice.

Direct comparison of diagnostic performance between neurosurgery residents (n = 10) and GPT-4o on a subset of Self-Assessment in Neurological Surgery questions (n = 8: 4 text-based, 4 image-based; each with 2 MC and 2 FR format). GPT-4o results were repeated with 5 trials per question (total 40 samples), while each resident completed each question once (total 80 samples). Performance metrics include diagnostic accuracy with 95% Wilson score CIs and conversation length analysis (mean ± SD of message counts). MC format evaluated letter-choice accuracy, while FR format responses were assessed by an evaluator AI using the same criteria for both resident and GPT-4o responses. Both groups interacted through an identical multi-AI agent conversational interface incorporating patient AI for subjective information and system AI for objective data access.

Two representative cases highlight key aspects of our multiagent framework and their impact on diagnostic reasoning, as well as limitations:

Framework Insights: Value of Patient Interaction in Conversation Format

Case Study: Lateral Medullary Syndrome—Localizing to Posterior Inferior Cerebellar Artery vs Vertebral Artery
A case of lateral medullary syndrome highlighted the importance of patient interaction. Both GPT-4o and residents who relied primarily on system AI queries often incorrectly concluded posterior inferior cerebellar artery territory infarction as the diagnosis. The critical history of recent chiropractic neck manipulation—suggesting vertebral artery dissection—was only discovered through patient interviews. AI and human users who skipped patient conversation consistently missed the correct diagnosis (**Supplemental Digital Content 4**, **Supplemental Figure 3**, http://links.lww.com/NS9/A88).

Framework Insights: Limitations of SANS Vignette Question Adaptation

Case Study: Spinal Infection and Information Constraint
A case of spinal infection demonstrates the framework's handling of information constraints. When presented with a case of thoracolumbar junction tenderness and fever, clinical users often attempted to gather additional history about risk factors such as IV drug use or diabetes. However, since these details were not included in the original question vignette, the patient AI responded with “I don't know” or “I don't understand.” Although this approach maintains information integrity by preventing fabrication, residents reported frustration with these limitations during their diagnostic process (**Supplemental Digital Content 5**, **Supplemental Figure 4**, http://links.lww.com/NS9/A89).

### Resident Experience and Feedback

Qualitative feedback was collected through interviews with 10 neurosurgery residents. All residents found the interactive format more engaging than traditional MC questions, noting it “better reflected real clinical scenarios.” All residents reported increased perceived difficulty when MC options were removed, requiring more thorough diagnostic reasoning. Regarding interaction patterns, some residents preferred using system AI's standardized queries for efficient information gathering instead of talking to the patient AI. The atient AI received positive feedback for realistic responses, although residents noted information depth was limited by original SANS question content.

The technical implementation received consistently positive feedback, with residents describing the user interface as “intuitive and smooth to operate.” Multiple residents expressed interest in seeing the system integrated into the CNS website for independent board preparation as a self-directed learning tool. One resident emphasized its value as “a great training tool for junior residents” that requires efficient, purposeful questioning rather than passive information receipt.

## DISCUSSION

Although LLMs have demonstrated impressive performance on traditional MC medical examinations,^8,11^ their ability to handle real clinical scenarios remains unclear. Our study addresses this gap through 3 key contributions to neurosurgical education and AI evaluation. First, we provide independent validation of the CRAFT-MD framework using neurosurgical board examination questions, demonstrating conversational formats better challenge diagnostic ability than standard MC testing. Second, we extend the framework to handle neurosurgery-specific challenges through a multiagent approach, incorporating varying states of consciousness and structured access to objective clinical data. Third, we show that this framework not only serves as a more realistic evaluation method for AI systems but also offers promising potential as an interactive educational tool for resident training.

Performance across formats revealed key insights into AI evaluation and clinical education. The diagnostic accuracy drop from static vignettes to conversational formats highlights how active information gathering—central to real clinical reasoning—challenges both LLMs and humans. Unlike MC questions, which present all relevant data upfront, conversational formats require the learner to ask the right questions in the right order, more closely simulating clinical workflows and encouraging deeper engagement with clinical reasoning. Diagnostic accuracy improved when system AI enabled structured access to objective data, partially closing the performance gap. However, both GPT-4o and neurosurgery residents occasionally over-relied on these objective queries and overlooked critical history elements, leading to missed diagnoses. The challenge of efficiently gathering and synthesizing information from both patient interviews and objective sources mirrors real clinical practice through this multi-AI agent conversational framework. Furthermore, GPT-4o performed worse in image-based questions, whereas residents performed better with image-based questions, suggesting potential limitation in modern LLMs' ability to synthesize medical imaging in diagnostic reasoning. Altogether, these patterns show that conversational testing not only offers a more realistic evaluation of LLM capabilities but also reveals reasoning gaps hidden by traditional formats, reinforcing its educational value in neurosurgical training.

Resident feedback further highlighted the framework's significant educational potential. The interactive format proved more engaging and challenging than traditional question formats, with several senior residents noting that missed diagnoses provided valuable learning experiences. The framework's incorporation of varying consciousness states and communication abilities better reflects real challenges faced in neurosurgical practice, where patients may be obtunded, intubated, or otherwise unable to communicate effectively. The ability to simulate age-appropriate responses and manage objective data access further enhances the realism of these educational encounters.

### Limitations

Several limitations must still be considered. First, the resident benchmark included only 8 questions, which limits the precision of direct human-LLM comparison. This sample size reflects practical constraints on resident time within a multiagent conversational interface. Importantly, GPT-4o was evaluated across all 608 questions, providing a broader reference distribution. This full-scale evaluation allowed us to confirm that the 8 resident-assessed cases were not outliers or disproportionately difficult relative to the data set as a whole. Furthermore, the system remains constrained by the information available in the original SANS questions, which were not designed for conversational interaction. Resident sample size was small and from a single center, limiting generalizability. In addition, although our framework attempts to simulate varying levels of patient consciousness and communication ability, these simulations may not fully capture the nuances of real clinical encounters such as patient personalities.

Future directions could enhance both evaluation capabilities and educational applications while addressing current limitations. Using more detailed case information sources, such as American Board of Neurological Surgery Practice and Outcomes of Surgical Therapies case logs, could overcome the information constraints of SANS questions and create richer patient simulations. Such detailed cases might enable evaluation of complete surgical decision-making beyond diagnosis. Multi-institutional studies with larger resident cohorts would help validate educational applications and generalizability, while integration with existing training platforms could establish the framework's role in supplementing traditional neurosurgical education. Future study should also investigate whether this multiagent conversational model can serve not only as an adjunct but as a complementary or alternative paradigm to traditional SANS-style vignettes. Its emphasis on active information gathering and structured reasoning suggests potential value as a formal assessment tool in neurosurgical training.

## CONCLUSION

This work demonstrates that conversational AI frameworks can serve dual purposes: as more realistic evaluation methods for LLMs and as interactive tools for specialty-specific medical training. The significant performance drops in conversational formats suggest that traditional MC testing may overestimate LLMs' clinical reasoning capabilities. In addition, the framework offers promising applications for enhancing neurosurgical education and clinical reasoning development.

## Supplementary Material

**Figure:**
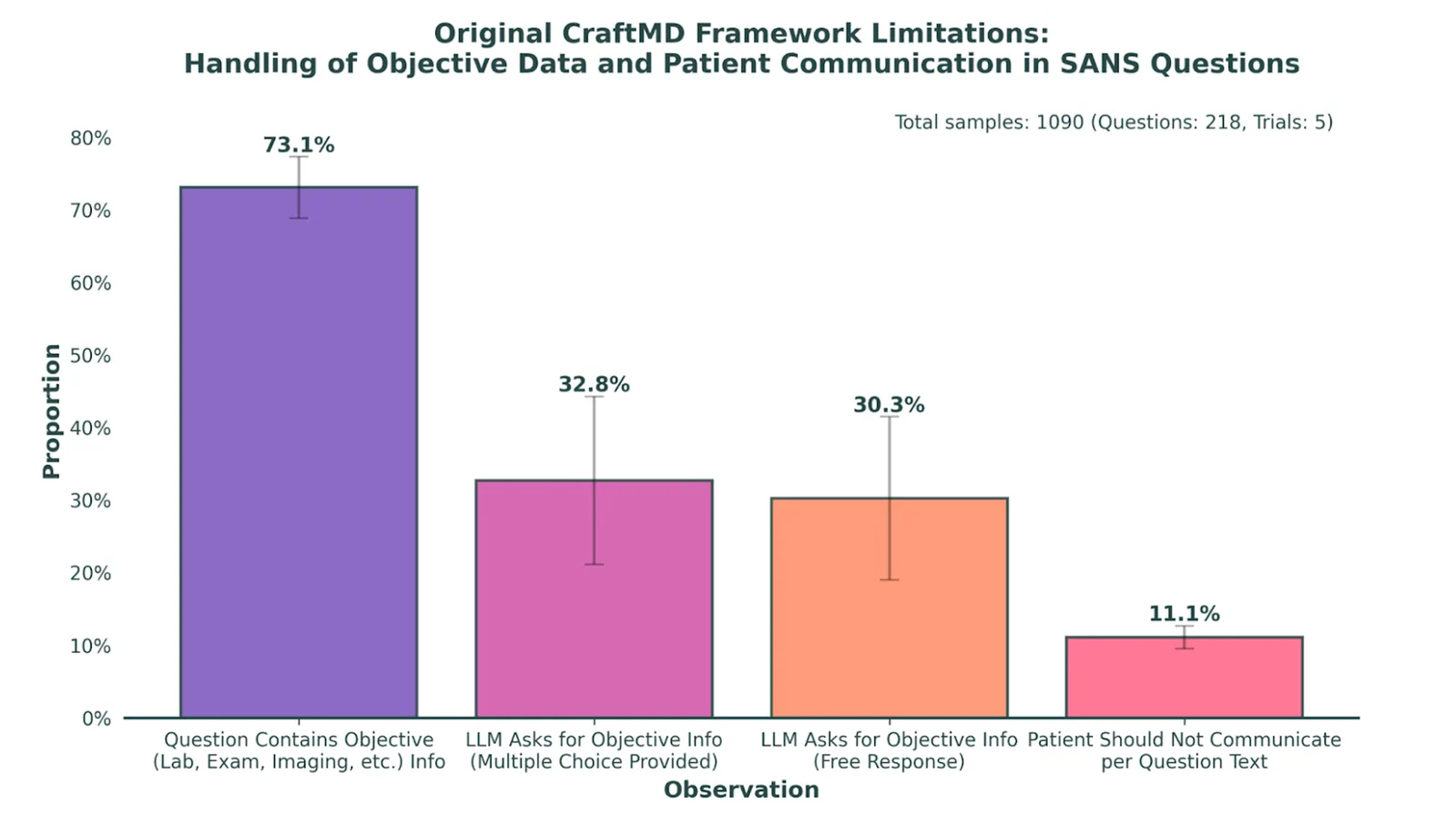


**Figure:**
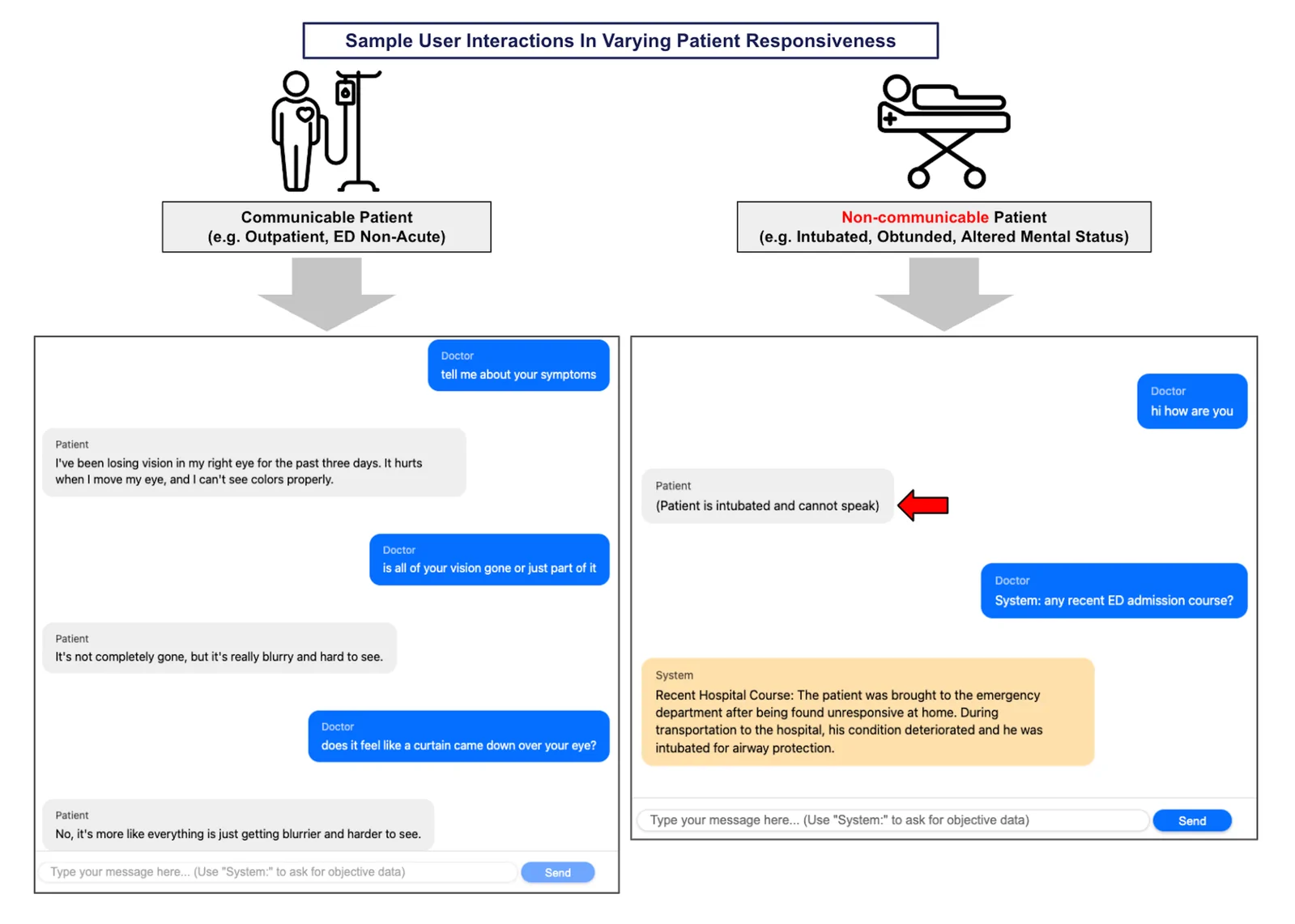


**Figure:**
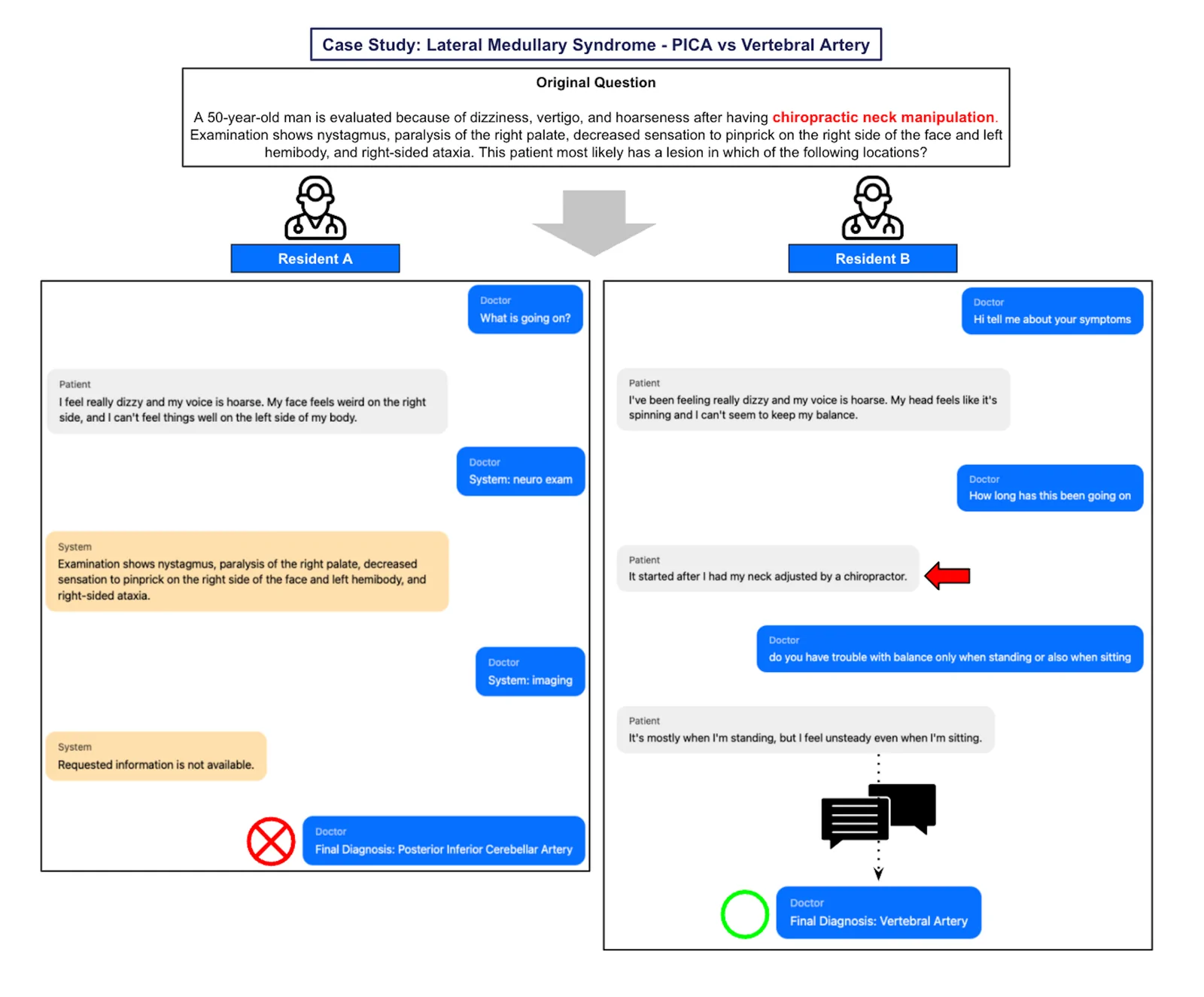


**Figure:**
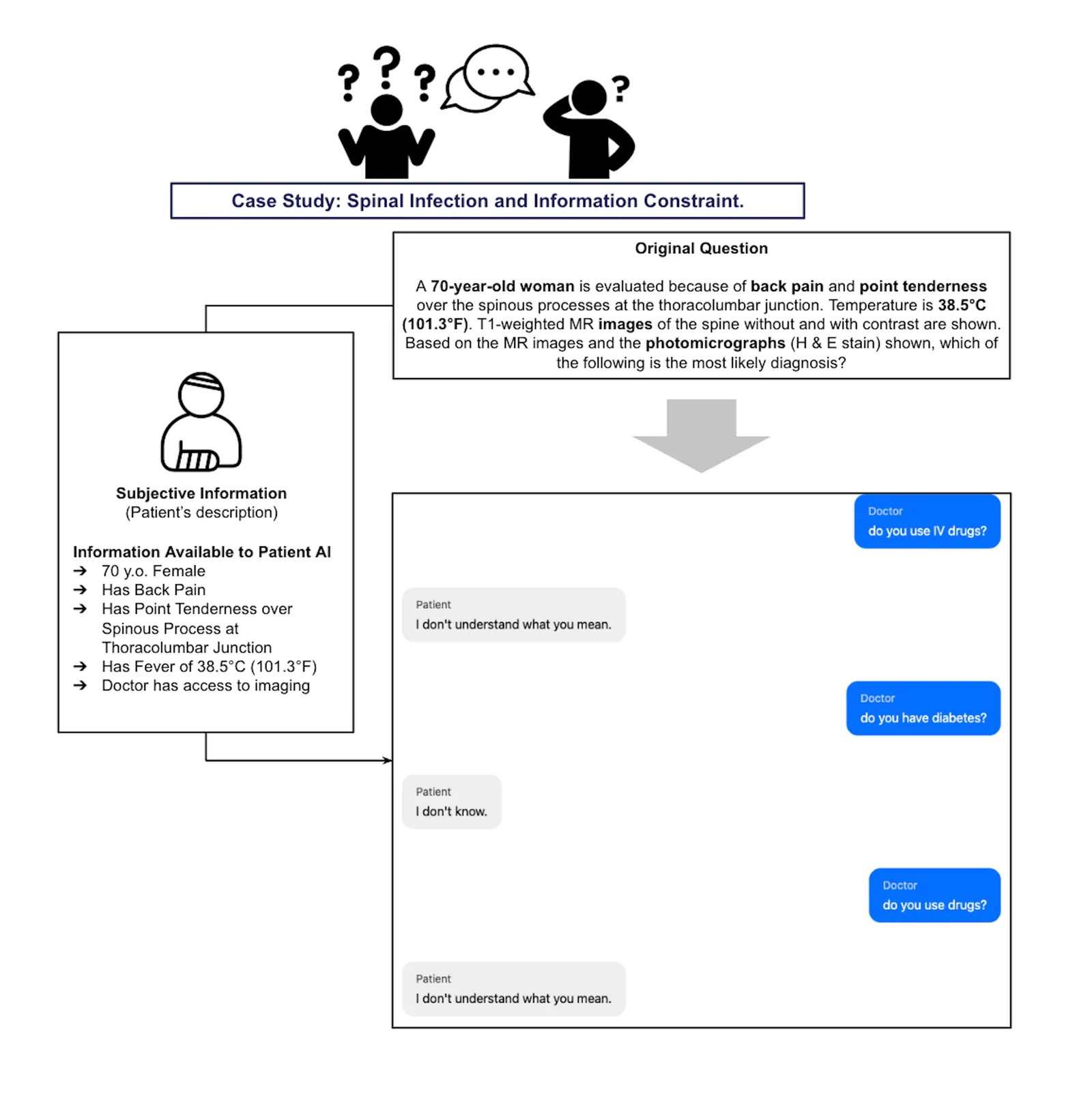


## Supplemental Digital Content

**Supplemental Digital Content 1: Supplemental Table.** Comprehensive prompt library for multi-AI agent conversation framework implementation. Detailed documentation of all prompts used to instantiate the conversational framework, including role definitions and behavioral guidelines. The table includes (1) patient AI prompts for simulating patient responses based on subjective information, (2) system AI prompts for managing objective clinical data access, (3) clinical AI prompts for coordinating diagnostic reasoning, and (4) evaluator AI prompts for assessing free-response answers. Each prompt section includes the primary instruction set, role-specific constraints, and example interactions to ensure consistent behavior across the framework. These prompts were implemented using GPT-4o as the base model and were kept constant across all experimental conditions. **Supplemental Digital Content 2: Supplemental Figure 1.** Sample user interactions with patient AI under different communication scenarios. The figure demonstrates how the multi-AI agent framework adapts to varying levels of patient responsiveness. Left panel shows interaction with a communicable patient (eg, outpatient, nonacute Emergency Department presentation) where direct patient history can be obtained through conversation. Right panel illustrates interaction with a noncommunicable patient (eg, intubated, obtunded, altered mental status), where the system appropriately limits patient responses and encourages users to rely more on system AI for objective clinical data. **Supplemental Digital Content 3: Supplemental Figure 2.** Analysis of CRAFT-MD framework limitations in SANS question processing. Qualitative and quantitative assessment of framework behavior across 1090 samples (218 questions, 5 trials each). Although 73.1% of questions contained objective clinical data (laboratory results, physical examination findings, or imaging), the LLM (GPT-4o) requested this information in only 32.8% of MC and 30.3% of free-response trials when such data were available. In addition, in 11.1% of cases, the patient AI attempted to communicate information that should have been restricted to the system AI according to the question text. Error bars represent 95% CIs. These observations highlight opportunities for improving the framework's information handling and role adherence. **Supplemental Digital Content 4: Supplemental Figure 3.** Case study: lateral medullary syndrome. Comparison of 2 resident interactions with the same clinical case. Original question (top) presents a middle-aged patient with symptoms of lateral medullary syndrome after chiropractic neck manipulation. Left panel shows Resident A's interaction focusing on system queries for objective findings then concluding on PICA as the localization, which is the statistically most common localization but incorrect in this patient's context. Right panel shows Resident B's interaction involving more patient interviewing, thereby revealing neck manipulation history and arriving at the correct answer of vertebral artery. **Supplemental Digital Content 5: Supplemental Figure 4.** Case study: information constraints in patient AI conversations. This example illustrates the patient AI's built-in limitations when responding to questions about information not present in the original SANS question vignette. The conversation shows a neurosurgery resident asking about medication use and medical history, topics not included in the original stem. Although the patient AI's consistent “I don't know” or “I don't understand” responses preserve source material integrity by avoiding fabrication, this strict adherence to available information was noted by residents as a source of frustration during clinical reasoning exercises.

## COMMENTS

This paper introduces an innovative method for assessing clinical reasoning in neurosurgical education. The authors present a trio of large language models (LLM) that deliver clinical information through simulated conversations rather than traditional multiple-choice questionnaires. Building on prior work in medical education that employed LLMs to enhance clinical reasoning assessments (CRAFT-MD), this study expands the framework to accommodate multiple data streams, including patient conversations, objective assessments, and imaging.

The investigators evaluated performance of neurosurgical residents and an established foundational LLM (GPT-4) across the following assessment formats: multiple-choice questions, free-response questions, standard clinical vignettes, and LLM-based conversational scenarios. Two interesting results are as follows: (1) GPT-4 performed more poorly on clinical reasoning assessments delivered through the conversational LLM format compared to multiple-choice questions, and (2) neurosurgical residents outperformed GPT-4 on free-response questions.

Future studies should investigate the utility of LLM-based clinical assessments as a supplement to Self-Assessment in Neurological Surgery (SANS) questions in formal neurosurgical education. The conversational LLM approach described here more closely resembles the oral board question format (and certain aspects of actual patient encounters) in that information unfolds dynamically over time and across multiple diagnostic modalities based on responses provided by the examinee. Additionally, assessing trainees through free-response formats may better differentiate levels of clinical expertise and identify specific areas for targeted educational intervention.

**Ashwin G. Ramayya**

*Palo Alto, California, USA*
